# Affect before diagnosis: applying affective neuroscience to psychiatry

**DOI:** 10.3389/fpsyt.2026.1858824

**Published:** 2026-06-12

**Authors:** John White

**Affiliations:** Texas Tech University Health Sciences Center School of Medicine, Amarillo, TX, United States

**Keywords:** Acceptance and Commitment Therapy (ACT), affective neuroscience, comorbidity, emotion regulation, personality, psychopathology, subcortical affective systems, transdiagnostic

## Abstract

Jaak Panksepp spent nearly five decades mapping the primary-process affective systems of the mammalian brain across different species, producing a framework of considerable empirical power that is functionally invisible within psychiatry. Psychiatry has not built upon that literature in human contexts. Consequently, Affect has never occupied the foundational place in psychiatry that the evidence warrants. This paper attempts to close that gap and assembles twelve converging lines of evidence for subcortical primacy of Affect, including evidence not previously synthesized for this purpose: pseudobulbar affect, gelastic and dacrystic epilepsy, double dissociation of volitional and emotional facial expression, affective blindsight, and neonatal emotional behavior. The evidence shows that Affect is generated subcortically, the cortex modulates rather than creates it, and when cortical regulation is removed or impaired, affective states persist or intensify. From this evidence, personality is best understood as an individual’s position in a configuration space defined by subcortical Affect generation parameters and cortical regulatory capacity, with psychopathology occurring when signal intensity chronically exceeds or overwhelms regulatory capacity. This framework generates a specific clinical prediction: conditions that co-occur with personality pathology at rates incompatible with independent etiology are expressions of the same affective architecture through different conditioned channels, not independent diseases. Evidence confirms this directly: when personality pathology improves, its comorbid conditions decline substantially; when it does not, they persist. Further, the affective-regulatory framework transforms the clinical encounter by replacing a character verdict with a neurobiologically grounded account in which regulatory capacity is buildable and recovery is construction rather than correction.

## The comorbidity problem

Every psychiatry resident begins training ready and eager, only to find themselves sitting across from a patient carrying five DSM diagnoses and recognizing, with growing unease, that what they are looking at is not five diseases but one person. Heritability studies consistently find shared genetic architectures across supposedly independent diagnoses (1, 2). A powerful predictor of any single disorder is the presence of another (3, 4). Something is fundamentally wrong with the map.

The diagnostic fragmentation is an artifact of the instrument, not a property of the patient. Comorbidity is the rule in this system rather than the exception, and the DSM describes the shape of suffering; it does not explain its origin. A nosology built on phenomenological description will inevitably classify by surface rather than by substrate. A cough is a cough whether it comes from tuberculosis, heart failure, or aspiration. Treating the cough without identifying its cause produces outcomes exactly as disappointing as treating depression, anxiety, personality pathology, somatization, and substance use in the same patient as categorically distinct conditions. This problem has been identified before. Psychiatry will not come of age as a medical discipline while disorders are addressed through outward appearances (5). Symptom-based categories without respect for any diagnostic hierarchy misattribute one condition to multiple categorical labels (6).

Nowhere is this more apparent than in personality pathology. The DSM personality disorder categories demonstrate consistently poor discriminant validity, enormous within-category heterogeneity, and comorbidity rates that approach absurdity (7, 8). It is common for individuals to meet criteria for three or more supposedly distinct personality disorders simultaneously (9). The Alternative Model for Personality Disorders in DSM-5 represents a meaningful step toward dimensional conceptualization (10), but it remains insufficiently grounded in the neuroscience that would give its dimensions mechanistic content.

The clinical reality is more telling still because personality pathology rarely presents alone. Dissociative symptoms (11), disordered eating (12), somatic and functional presentations (13, 14), and sustained substance use (15, 16) co-occur with personality dysfunction at rates that categorical models cannot explain without invoking coincidence. Some patients dissociate. Some cut. Some purge. Some drink. Some shake. Some have it all. The DSM treats each as an independent condition requiring its own etiological explanation and as separate from the personality disorder. Clinical experience suggests these are not independent conditions.

This paper argues that Jaak Panksepp’s Affective Neuroscience framework provides the mechanistic foundation needed to reconceptualize personality and the broader domains of psychopathology it generates. Affective Neuroscience in its broadest form is any neuroscientific investigation of emotional processes. In its Pankseppian form, which is the version this paper draws on, it refers specifically to the program of research that mapped the primary-process affective systems of the subcortical mammalian brain.

Rather than beginning with behavioral symptom clusters and reasoning backward toward mechanism, this paper begins with mechanism and reasons forward toward behavior. The core proposition is parsimonious: personality, both normal and pathological, is best understood as an individual’s position in a configuration space defined by two interacting variables: the intensity of affective signal generation in subcortical circuits, and the capacity of cortical regulatory systems to modulate, contextualize, and redirect those signals. What transforms this simple framework into a generator of diverse clinical presentations is not additional theoretical complexity, but the combinatorial architecture of the systems involved.

## Affective neuroscience: the framework

Jaak Panksepp (1943–2017) spent nearly five decades mapping the primary-process emotional systems of the mammalian brain using lesion studies, deep brain stimulation, pharmacological manipulation, and direct neurochemical measurement across multiple species. He coined the term Affective Neuroscience in a 1992 paper and elaborated the framework in his seminal 1998 text of the same name (17, 18). His central claims can be stated precisely: there are evolutionarily conserved, subcortically generated affective states shared across mammals that are primary and not derived from cognition, and that drive behavior in ways that precede and partially determine conscious experience (18).

Panksepp always wrote Affects in capitals to distinguish them from folk-psychological uses of the same terms. The positive valence systems drive mammals toward what they need. SEEKING generates the exploratory urge that compels an organism to engage with its environment, to forage, to investigate, to pursue goals whose outcome is not yet known. Without it, no mammal would leave the nest. LUST ensures reproductive behavior occurs with sufficient motivational force to overcome the costs and risks of mating. CARE binds parent to offspring with enough strength to sustain the prolonged period of dependence that mammalian development requires. PLAY, observed most prominently in juveniles, drives the rough-and-tumble social engagement through which young mammals learn the boundaries of acceptable social behavior, a calibration process without which adult social competence does not reliably develop. The negative valence systems protect mammals from what can destroy them. FEAR mobilizes escape and avoidance in the presence of predatory or environmental threat. RAGE is activated when an organism’s access to resources or freedom of action is blocked, generating the aggressive arousal needed to overcome obstacles and defend what has been secured. GRIEF/PANIC is the separation distress signal, the powerful aversive state that arises when social bonds are ruptured and that motivates the urgent reestablishment of contact with attachment figures, a system without which no socially dependent mammal would survive infancy.

Each of the seven systems operates with its own characteristic neurochemistry, its own anatomical substrate, its own pattern of autonomic and motor output, and its own valence signature. Electrical stimulation of specific medial brainstem and hypothalamic regions reliably evokes coherent, directional affective states in animals (19, 20). Lower current is required to evoke emotional responses from subcortical structures than from cortical ones. This demonstrates that the circuitry is more concentrated and sensitive at lower levels. Animals given the opportunity to self-stimulate positive valence circuits do so avidly; animals given the opportunity to terminate negative valence stimulation do so immediately. This is not learned behavior. This can only be the expression of pre-wired valenced response to direct neural activation (18).

A full treatment of these systems exists in Panksepp’s primary texts (18, 21) and in Davis and Montag’s clinical summary (22), and is not reproduced here. For the present argument, the seven systems are taken as established, not because the framework is beyond scrutiny, but because the core empirical findings on which it rests have not been successfully refuted in three decades. What matters for this paper is the framework’s central architectural claim: Affect is generated subcortically, operates prior to and independent of cortical processing, and the cortex’s primary role in emotional life is modulatory rather than generative.

## The evidence for subcortical primacy of affect

The claim that Affect is generated subcortically and modulated cortically rests on multiple, diverse, and converging lines of evidence. What follows assembles these for a clinical audience, as several have not been considered for this purpose and none have been adequately accounted for in clinical thinking about personality and psychopathology.

Decortication: Beginning with Bard’s demonstration of sham rage in cats in 1928 (23), the decortication literature provides the most direct experimental evidence for the central premise of this paper. Animals whose cortex has been surgically removed retain the full affective behavioral repertoire. They orient to stimuli, pursue goals, and engage in coordinated motivated behavior involving integration of information and selection among behavioral options. Decorticate cats exhibit extreme rage reactions to mild stimuli that would barely register in an intact animal. Decorticate rats engage in rough-and-tumble play virtually indistinguishable from intact animals in overall vigor (24). Panksepp tested this directly. In a laboratory practicum, sixteen graduate students observed pairs of rats for thirty minutes, one decorticated at three days of age, one a sham-surgery control, and were asked to identify which animal lacked a cortex. Twelve of the sixteen chose wrong. The consistent reasoning among those who erred was that the decorticate animal appeared more engaged, more exploratory, more interested in the world. They mistook disinhibited subcortical SEEKING for neurological normality. The animal running without a cortex looked, to trained observers, like the more alive one (25). That is precisely the point. Remove cortical inhibition and Affect does not disappear. It runs harder without a brake.

Additional examples span classical conditioning in decorticate rabbits (26) and dogs (27), and complex spatial behavior in decorticate rats (28). The implications are unambiguous. Primary affective states do not require cortical processing. The decortication literature is the strongest and most well-replicated body of evidence bearing on this question, and it has not been appropriately accounted for in clinical practice. There is no reason to believe decorticate humans would be exempt from mammalian physiology, and as the following sections demonstrate, we are most certainly not.

Hydranencephaly: A condition in which the cerebral hemispheres are largely or entirely replaced by cerebrospinal fluid while subcortical structures remain intact. Children born with hydranencephaly are routinely assigned a vegetative state on the assumption that without a cortex there can be no consciousness. Merker’s 2007 review documented systematic behavioral evidence to the contrary (29). Hydranencephalic children display emotional expressivity including smiling, laughing, and crying. They orient to stimuli. They recognize caregivers and display differential responses to familiar versus unfamiliar people. They show clear evidence of preferences, distress, and comfort. The primary methodological caveat is that radiographic confirmation of complete cortical absence is imperfect, and some cases may retain residual tissue, a limitation Merker acknowledged. Given the ethical impossibility of experimental decortication in humans, this represents the closest available human analog to the animal literature, and the behavioral profile it documents is what mammalian decortication research predicts.

Neonatal emotional behavior: Just ask any new parent or third-year medical student surviving their OB/GYN clerkship, newborns display organized emotional responses. From the first hours of life they demonstrate distress, comfort, orienting, and eventually differential responses to familiar versus unfamiliar people. Newborns do this before cortical motor outflow exists. Cortical control of eye, face, and limb movements does not emerge until months after birth; neonatal motor behavior is generated by brainstem networks (30). If the cortex cannot produce movement in neonates, neonatal emotional expression is necessarily subcortically generated. Human infants arrive with a full affective repertoire months before the cortex is functionally capable of producing any of it (31).

Pseudobulbar affect: PBA occurs when cortical inhibitory pathways to subcortical emotional expression circuits are damaged by ALS, MS, stroke, TBI, Parkinson’s disease, or any other process disrupting descending corticobulbar fibers (32). Patients display brief, intense episodes of crying or laughing that are mood incongruent and triggered by trivial or neutral stimuli. A patient with PBA may laugh uncontrollably when angry and frustrated. This is the decortication phenomenon in living humans. Subcortical affect generation is intact, the cortical braking system is damaged, and raw bottom-up affective expression runs without constraint.

The pharmacology is consistent with the mechanism. PBA responds to SSRIs (33), dextromethorphan-quinidine (34), and levodopa (35), none of which act primarily on cortical cognition, and all of which modulate the neurotransmitter systems implicated in subcortical emotional expression circuits. Notably, the therapeutic effect of SSRIs in PBA appears independent of their antidepressant action and occurs more rapidly, indicating a distinct mechanism operating below the level of cortical neuroplastic change (33). In Parkinson’s disease, PBA can present as a levodopa off phenomenon. When dopaminergic tone drops, cortical inhibitory control of subcortical affective expression circuits is released, producing PBA as a non-motor off phenomenon. Restoring levodopa restores that inhibitory control (36).

Gelastic and dacrystic epilepsy: Focal seizures originating in the hypothalamus, usually associated with hypothalamic hamartomas, produce paroxysmal laughter in the gelastic form and paroxysmal crying in the dacrystic (37, 38). Patients occasionally report genuine mirth during gelastic seizures and felt sadness during dacrystic ones, suggesting that hypothalamic circuitry can instantiate not merely emotional behavior but primary affective experience itself.

Deep brain stimulation: DBS provides something close to the controlled activation equivalent of animal electrical stimulation experiments in humans. Subthalamic nucleus DBS has induced pseudobulbar crying and mirthful laughter in Parkinson’s patients with no prior history of such episodes (39, 40). Patients in the laughter cases report genuine mirth accompanying the stimulation-induced response, while crying cases occur without subjective sadness, together demonstrating that subcortical stimulation can both generate primary affective experience and produce emotional expression dissociated from cortical mood state. The STN sits at the intersection of motor, cognitive, and limbic circuits, and its pathological hyperactivity in Parkinson’s disease produces not only motor inhibition but emotional inhibition as well. DBS deafferentation of the STN releases that brake, allowing both motor and affective disinhibition (41). This is direct subcortical stimulation producing coherent, valenced affective states in awake humans, independent of any cortical processing initiating it.

Cortical stimulation: Over decades of intraoperative stimulation of the exposed cortex in awake patients, Penfield reliably produced sensory experiences, involuntary movements, and vivid memory fragments (42). What he did not reliably produce was felt emotion. A comprehensive review of one hundred years of human electrical brain stimulation confirms the asymmetry: cortical stimulation across frontal, parietal, temporal, and occipital regions predominantly evokes sensory, motor, and cognitive phenomena, while coherent affective states are evoked most reliably from subcortical stimulation (43). Panksepp drew the logical inference: if the cortex generated Affect, direct stimulation should evoke it, and it does not (44). The evidence points in one direction. Affect is generated subcortically.

Double dissociation; facial paresis: Patients with volitional facial paresis from cortical stroke retain the ability to smile and laugh spontaneously when genuinely amused. Conversely, patients with lesions of subcortical and deep limbic structures can smile voluntarily on command but cannot produce spontaneous emotional expressions when subjectively feeling amused (45, 46). This double dissociation directly demonstrates that emotional expression has a subcortical generator that is anatomically and functionally independent of cortical control.

Double dissociation; dreaming: Solms demonstrated another neat example of double dissociation. Patients with brainstem lesions that eliminated REM sleep continued to dream, while patients with focal lesions disrupting the mesolimbic dopaminergic pathway corresponding to Panksepp’s SEEKING system stopped dreaming entirely while retaining normal REM architecture (47). The cessation of dreaming following prefrontal leucotomy has been documented in nearly 1,000 cases (48). Dreaming is enhanced by dopamine agonists and suppressed by antagonists with no change in REM frequency (49). The brain generates an entire world of conscious affective experience during sleep, driven by subcortical circuitry, largely independent of cortical initiation.

Affective blindsight: Patients with destruction of the primary visual cortex cannot consciously see, yet they discriminate between emotional facial expressions presented to their blind field at above-chance rates (50). This is mediated by a subcortical pathway from the superior colliculus through the pulvinar to the amygdala, bypassing cortex entirely, and the pathway undergoes structural strengthening in blindsight patients (51). Critically, physiological responses to unseen emotional stimuli are faster and stronger when cortical processing is suppressed (52). This is consistent with the pattern described throughout this section: the cortex dampens subcortical affective signals rather than generating them.

Neuroimaging: Damasio’s PET imaging research found that the experience of feeling emotions reliably engaged upper brainstem nuclei, while cortical regions showed no such reliable relationship to felt affect (53). The regulatory neuroimaging literature reinforces the same architecture from the opposite direction. When cortical systems are recruited to modulate affective experience, the consistent finding is prefrontal activation paired with subcortical suppression. Cognitive reappraisal recruits prefrontal and parietal control regions while attenuating amygdala responses (54), and ventromedial prefrontal cortex and the amygdala are inversely coupled during negative affect regulation, with greater prefrontal engagement predicting greater amygdala suppression (55). Affect is generated below and regulated from above. A recurrent finding, the cortex is a brake, not an engine.

Evolutionary conservation: The mammalian midbrain architecture is conserved across species that diverged hundreds of millions of years ago. If emotional systems were cognitive constructs requiring extensive cortical processing, they would vary with cortical complexity. They do not. The seven systems are recognizable across species with radically different cortical architectures, in precisely the regions evolution has most conserved. Darwin made this argument from phylogeny in his aptly titled *The Expression of the Emotions in Man and Animals* (56). Panksepp spent fifty years providing the neural specificity Darwin lacked (18).

Despite the depth and breadth of Panksepp’s empirical program, its implications for clinical psychiatry have remained largely unaddressed. An examination of his publication record is instructive. His work appeared in neuroscience, physiology, behavioral, psychobiological, and psychopharmacology journals. It did not appear in the clinical outlets where practicing psychiatrists encounter new ideas. This was not an accident of interest. Panksepp was explicitly motivated by psychiatric applications throughout his career and made increasingly direct moves in that direction in his final decade. The clinical fields did not meet him halfway. The explanation lies elsewhere: the fields most relevant to understanding and treating personality and psychopathology occupy separate and rarely overlapping publication ecosystems. Neuroscientists publish in neuroscience journals. Research psychologists publish in psychology journals. Personality researchers publish in personality journals. Psychiatrists publish in psychiatry journals. Each field attends different conferences, reads different literature, and operates within different theoretical traditions. The result is a framework of considerable empirical power that has remained functionally invisible to the clinicians who need it most.

Panksepp understood the methodological constraints his program operated under. The deep subcortical structures he studied are inaccessible to the non-invasive methods available in humans, and the direct manipulations that established the seven systems required animal models. He accepted this as a feature of rigorous science, not a limitation to apologize for. The mammalian brain is conserved across species in precisely the regions he investigated, and the behavioral and neurochemical homologies between non-human mammalian and human affective systems are not speculative. They are among the most well-replicated findings in comparative neuroscience. The appropriate response to this methodological reality is not to dismiss the animal literature but to build upon it carefully in human contexts, using the converging lines of evidence that are available. That has not happened. The subcortical affective systems have been largely ignored by clinical psychiatry, psychology, and clinical neuroscience, and Affect has consequently never occupied the foundational place in psychiatric theory that the evidence warrants. The gap is not methodological. It is institutional and conceptual.

Even though Panksepp’s empirical program was conducted almost entirely in non-human animals, the lines of evidence assembled in this section demonstrate that the same principles apply in humans. Direct neural manipulation, neuroimaging, surgical ablation, congenital absence of cortex, acquired cortical disconnection, seizure activation, and evolutionary conservation all point to the same principle. Affect is generated subcortically, the cortex modulates rather than creates it, and when cortical regulation is removed or bypassed, affective states persist or intensify. This is the foundation on which a mechanistic account of personality and psychopathology can be built.

## Affective-regulatory configuration

Panksepp proposed that the brain processes affective information across three nested levels. The evolutionary logic is essential. The cortex is new. The midbrain is ancient. The direction of influence flows primarily bottom-up. Primary processes are the subcortical affective systems themselves. These are instinctual, evolutionarily ancient, and operating below conscious awareness. Primary processes are densely interconnected, mutually modulating, and capable of simultaneous activation and of combinations that produce states far more complex than any single system generates alone.

The primary-process systems do not project their outputs into a void. They feed upward into secondary-process mechanisms, the learning and memory systems. Secondary processes are mediated primarily by the basal ganglia, extended amygdala, and associated limbic regions, that connect raw Affect to the world through classical conditioning, operant learning, and emotional habit formation (18, 57, 58). At this level, a context-free FEAR signal becomes a specific phobia, a trauma-linked flashback, or a conditioned avoidance pattern (59). A primary SEEKING surge becomes attached to a substance (60) or gambling behavior.

**Figure 1:**
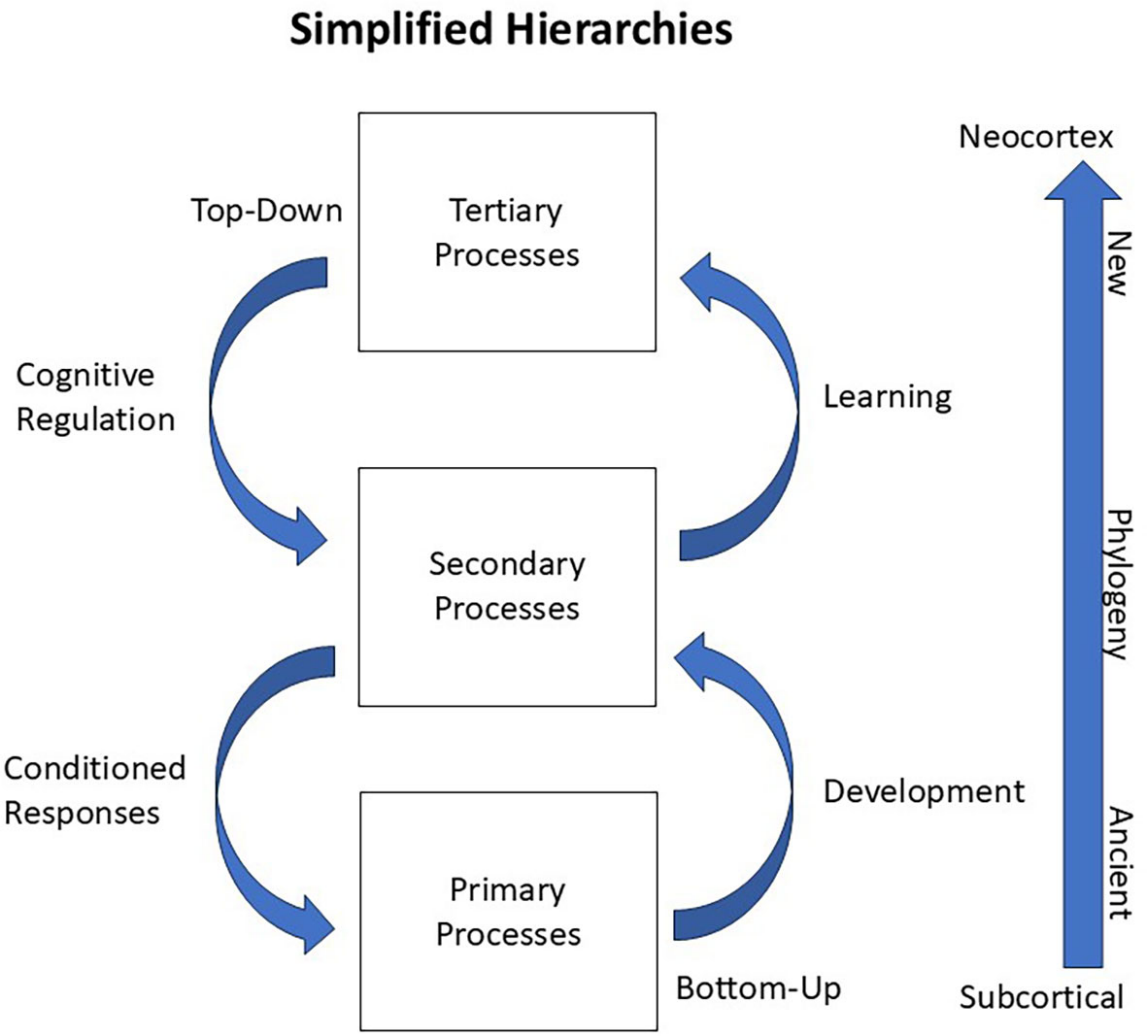
Simplified hierarchy of primary, secondary, and tertiary processes across phylogenetic levels. Adapted from Panksepp, J., & Davis, K. L. (2018). The Emotional Foundations of Personality. Norton.

Tertiary processes are neocortical elaborations, the thoughts, narratives, conscious regulatory strategies, and deliberate modulation of Affect through prefrontal regions (21). The prefrontal regulatory system is not a single brake. The medial prefrontal cortex, orbitofrontal cortex, dorsolateral prefrontal cortex, and anterior cingulate cortex each contribute distinct regulatory functions, from the automatic, implicit regulation of ventromedial prefrontal circuits to the effortful suppression and reappraisal strategies mediated by dorsolateral regions (61, 62). The anterior cingulate monitors conflict between affective impulses and regulatory goals, recruiting additional prefrontal resources when the mismatch exceeds a threshold (63). An individual can have intact deliberate reappraisal capacity and severely compromised automatic regulation, or the reverse. **Figure 1** illustrates the simplified hierarchy of primary, secondary, and tertiary processes across phylogenetic levels.

This position has precedent in the personality disorder literature. In Kernberg’s *What is personality?* he explicitly identified Panksepp’s primary affective systems as the constitutional foundation of temperament, which he treated as the motivational bedrock of the entire personality structure (64). He then built on that bedrock with psychoanalytic constructs and theory, elaborating personality almost entirely in psychoanalytic terms. Object relations, splitting, projective identification, superego layering, and identity diffusion become the primary explanatory apparatus. The neuroscience recedes. Kernberg does maintain Affect as an ongoing motivational force throughout his account, but his elaboration moves away from neuroscience rather than toward it, and in doing so loses the most important implication of Panksepp’s framework.

Subcortical affective systems are not merely developmental substrates that organize into higher-order structures and gradually recede in importance. They run continuously beneath whatever cortical organization has developed. They are always present, always driving behavior, always waiting to be aroused, always the primary motivational force in a mammalian organism. When regulatory capacity is overwhelmed or intentionally disregarded, Affects make themselves known with considerable force. Kernberg’s psychoanalytic architecture captures something real that is universally accepted in development: early affective experience shapes personality structure. His further elaboration does not capture the neuroscience. Kernberg had Panksepp available to him and still chose the intellectual traditions of his field. Freud had no such choice.

The elaboration of personality in psychoanalytic terms represents not a failure of observation but a failure of multidisciplinary integration to follow the neuroscience where it leads. Interestingly, others have recognized the importance of affective neuroscience as a foundation for new theoretical frameworks. Unfortunately, due to the weight of tradition the urge to graft unfalsifiable constructs onto an empirical substrate in ways that undermine the very rigor the neuroscience provides is too hard to resist. The recently put forth ARCH model, for example, grounds behavior in evolutionarily conserved neural systems and explicitly cites Panksepp as foundational, yet superimposes Jungian archetypal constructs that lack equivalent empirical grounding, producing a framework that is more elaborate but less falsifiable than the affective neuroscience on which it depends (65). The deeper problem is one of direction. Psychology while beginning to recognize the importance of Panksepp’s work has largely attempted to accommodate affective neuroscience within existing cortex-first frameworks, treating subcortical Affect as an input to higher-order cognitive construction rather than as the primary generative force. What Panksepp’s program actually demands is the opposite: building cognitive and clinical theory upward from subcortical Affect rather than retrofitting Affect into architectures designed without it.

Psychoanalysis was closer to the truth than its critics acknowledged and further from it than its adherents admitted. The core observations, that behavior is driven by forces outside awareness, that early relational experience is formative, that impulse and inhibition are in perpetual tension, map with reasonable fidelity onto primary-process Affect generation, secondary-process conditioning, and prefrontal regulatory capacity. What psychoanalysis lacked was not clinical acuity but mechanistic grounding. Had it followed the neuroscience rather than defending the metapsychology, it would have found many of its concepts not refuted but located. The subcortical affective systems begin to provide exactly that neuroanatomical address for what psychoanalysis spent a century describing functionally. Patients are driven by forces they do not understand, responding to emotional signals they cannot name, and behaving in ways inconsistent with their stated values and intentions. Substituting the word subcortical for subconscious does not solve all the problems of psychoanalytic theory, but it grounds the observation that matters most in identifiable neural circuitry (66). What psychoanalysis meant by unconscious was not only outside of awareness but purposive. The subconscious, in the original formulation, was a wanting thing. Panksepp’s primary affective systems are similarly motivational. SEEKING is directional by definition, and the others are no less so.

The word emotion itself derives from the Latin *emovere*, to move out, to set in motion. Embedded in the word is the observation that emotions are not passive internal states but motivational forces that move an organism to action. A clinical practice focused primarily on cortical processes, on cognitions, identity, narratives, and beliefs, is working one level above the substrate that matters most.

### Parametric variation in the affective-regulatory system

Within this architecture, personality and the psychopathology it generates can be defined with unusual precision. Personality is the relatively stable configuration of parameters across an individual’s subcortical affect-generating and cortical regulatory systems. At the level of primary-process generation, each of the seven affective systems varies across individuals along at least four continuously distributed dimensions, baseline tonic activity (emotional set point), phasic reactivity (amplitude and speed of response to triggering stimuli), recovery kinetics (rate of return to baseline following activation), and threshold sensitivity (intensity of stimulation required to elicit a response). These four parameters across seven systems yield 28 continuously varying dimensions at the primary-process level alone. The number of possible activation patterns across seven systems, each varying continuously in intensity, each capable of tonic or phasic firing, each interacting nonlinearly with the others, is for all practical purposes endless.

Secondary-process conditioning multiplies this configuration space enormously. Every learned association between a primary Affect and a specific trigger, context, or behavioral sequence represents an additional dimension along which individuals differ. Two people with identical primary-process FEAR reactivity can develop profoundly different behavioral profiles depending on the conditioning history that shapes their responses. An individual with elevated FEAR reactivity whose early environment was characterized by unpredictable parental anger probably develops a very different phenotype than an equivalently FEAR-reactive individual whose primary threat was social abandonment. The primary-process substrate is the same; the secondary-process landscape is entirely different. One may meet criteria for social anxiety disorder. The other for dependent personality disorder. Categorical systems treat these as unrelated conditions which implies there are distinct etiologies. The affective-regulatory framework reveals them as parametric neighbors in a continuous space, separated by the direction of learning rather than by any fundamental difference in mechanism.

Multiply seven primary systems, by the learned associations each acquires through conditioning, and then multiply again by the distinct regulatory capacities of multiple prefrontal subregions, each with its own developmental trajectory and vulnerability profile. The result is a configuration space of such dimensionality that it accounts naturally for what clinicians observe. Every patient is recognizably human, recognizably patterned, and yet irreducibly individual. It is no wonder that humans generally consider each individual as “unique”. This is what personality is. It is not a list of traits extracted from questionnaire data. It is an individual’s position in a high-dimensional affective-regulatory configuration space, partially heritable, partially shaped by developmental experience, partially stabilized by cortical feedback dynamics, and expressed behaviorally through the conditioned channels that learning has laid down.

This is not a speculative taxonomy. The Affective Neuroscience Personality Scales, developed by Davis and Panksepp, represent an established psychometric instrument anchoring personality measurement directly to primary-process emotional systems (67, 68). These scales are not a fringe instrument; they are part of active research programs with a growing literature (69, 70). They represent one of the first serious attempts to anchor personality measurement to what actually drives behavior, a human being’s primary-process affective systems.

The dominant empirical tradition in personality research has been, for the better part of a century, fundamentally descriptive. From Allport through Eysenck, Cattell, and the eventual convergence on the Five-Factor Model, the field has observed human behavior, solicited self-report, and subjected the resulting data to successive rounds of factor analysis. What no amount of trait-based factor analysis can provide is a mechanistic account of how personality arises and why individuals occupy the positions they do in a trait space. The affective-regulatory framework offers a mechanistic understanding. Traits are the observable statistical regularities that emerge when the underlying affective-regulatory parameters are stable enough to produce consistent behavioral patterns across time and context.

Personality pathology is not a categorical disease. It is an extreme region of the configuration space where the parameters of affective generation and cortical regulation produce sustained functional impairment. Borderline personality disorder, the most studied example, emerges naturally as a configuration characterized by high baseline reactivity across multiple primary-process systems, particularly RAGE, PANIC/GRIEF, and FEAR, combined with severely compromised prefrontal regulatory capacity. Developmental research consistently links this configuration to the interaction of temperamental vulnerability with early relational trauma (71, 72). The nine DSM-5 criteria for BPD are the behavioral surface of a single affective architecture. Unstable relationships reflect PANIC/GRIEF reactivity to separation distress, which at its most extreme can be perceived abandonment that overwhelms regulatory capacity. RAGE activation without prefrontal appraisal or modulation leads to affective dysregulation with extreme behaviors that lead to clinical attention. Impulsivity reflects SEEKING surges without prefrontal braking. Affective instability reflects high-amplitude oscillations without cortical damping. Chronic emptiness reflects SEEKING system depletion following repeated regulatory exhaustion. Identity disturbance is the downstream consequence of living in an affective environment that shifts too rapidly for a stable self-model to consolidate.

The extensive comorbidity between BPD and depression, anxiety disorders, PTSD, eating disorders, and substance use is not the coincidental overlap of independent diseases. Shah and Zanarini (73) document that mood disorders affect up to 96% of BPD patients at some point, anxiety disorders up to 88%, substance use disorders up to 84%, and eating disorders up to 53%. The DSM treats each as an independent condition. The affective-regulatory framework treats them as expressions of the same underlying configuration through different conditioned channels. The same combination of elevated affective reactivity and reduced regulatory capacity, shaped by a conditioning history organized around food-related emotional regulation, produces disordered eating. Shaped by substance-related conditioning, it produces addiction. Shaped by social-evaluative conditioning, it produces social anxiety. Shaped by few or many of these simultaneously, it produces the familiar clinical picture of a patient who meets criteria for a personality disorder, an eating disorder, a substance use disorder, and an anxiety disorder all at once. Critically, when BPD improves, these comorbid conditions decline substantially as well. When BPD does not improve, they remain stable (73, 74).

The Collaborative Longitudinal Personality Disorders Study, an independent NIMH-funded prospective cohort, examined whether changes in BPD and MDD predicted each other over three years of follow-up using two different analytic methods. The result was the same either way: improvement in BPD consistently preceded improvement in MDD, while improvement in MDD did not predict improvement in BPD. The authors controlled for medication status and treatment intensity, and neither changed the result (74). The same unidirectional pattern holds for anxiety disorders. A separate CLPS analysis found that improvement in BPD significantly predicted remission from both GAD and PTSD, while the course of those anxiety disorders had no effect on BPD remission or relapse (75). A further CLPS analysis followed 302 patients with current MDD for two years and found that those without any personality disorder remitted at 89%. Those with comorbid BPD remitted at 60%. BPD predicted slowed MDD remission even after accounting for every other negative prognostic factor the investigators could identify. Patients with personality disorders received more treatment than those without, and still remitted more slowly (76). The ten-year CLPS outcome data complete the picture. The MDD comparison group in that study was selected to exclude personality disorder, and 80% of those patients achieved remission within one year. The BPD group reached only 30% in the same period, a gap the investigators attributed directly to the presence of personality pathology (77). Personality pathology did not merely predict the presence of comorbid conditions. It determined their course.

The directionality of these findings should give clinicians pause. When the personality organization improves, the constellation of comorbid conditions improves with it. When it does not, they persist. This is not a subtle finding. It suggests that a system which allows patients to accumulate diagnostic labels while the underlying architecture goes unaddressed is not serving them well. Patients are not failing treatment. The conceptual framework is failing patients. It raises a genuinely difficult question: can a patient with significant personality pathology have depression as a discrete and independent condition, or is what clinicians are calling depression the affective surface of an architecture that categorical diagnosis was never built to see? If the latter, then treatment resistance may reflect not a refractory illness but a refractory framework. The personality organization is the foundation, not one condition among many.

The same affective-regulatory logic applies across the clinical domains that most reliably co-occur with personality pathology. Dissociative symptoms emerge when affective signals of sufficient intensity exceed the integrative capacity of tertiary-process systems responsible for constructing a coherent self-narrative, producing the subjective experiences of derealization, depersonalization, and discontinuities in memory and identity that define the clinical picture. Prefrontal dysfunction is the most prominent neuroimaging finding across dissociative disorders, with additional dysfunction in the anterior cingulate and altered connectivity between subcortical and cortical regions (78, 79). Clinically, dissociative phenomena appear most reliably in patients whose affective-regulatory architecture is already significantly dysregulated, most commonly those with histories of early relational trauma.

The eating disorder spectrum requires a distinction. Restrictive anorexia nervosa is a discrete clinical entity with a meaningfully different neurobiology (80), ego-syntonic symptomatology (81), and a mortality rate that demands its own clinical account (82); it should not be dissolved into a transdiagnostic framework that cannot accommodate it. Binge and binge-purge presentations are a different matter. Elevated primary-process Affect and impaired prefrontal modulation drive the recruitment of food-related behavior as an attempted regulatory channel, and personality dysfunction in these presentations is not an occasional comorbidity but the rule (12).

Somatic and functional presentations follow the same pattern. Patients with comorbid personality dysfunction frequently cycle through medical and neurological workups while the affective architecture driving their symptoms goes unaddressed (14). In sustained substance use, the co-occurrence of personality dysfunction is among the most consistent observations in addiction psychiatry, even when it is underappreciated or deferred because the neurobiological effects of intoxication and withdrawal complicate personality assessment (15, 16). In each domain, treating the downstream expression without addressing the underlying configuration is renovation without repair.

The clinical implications of this reframing extend beyond theoretical interest. There is a categorical difference between telling a patient they have a personality disorder and telling them that their affective systems generate signals of high intensity, that their prefrontal regulatory capacity is underdeveloped relative to the demands placed on it, and that both of these parameters can change. Patients given a neurobiologically grounded account of how their affective systems work, why they feel what they feel, and why certain triggers produce disproportionate responses frequently describe a sense of recognition and relief that precedes and facilitates therapeutic engagement. Understanding that one’s affective reactivity reflects identifiable neural parameters rather than moral failure or irremediable character defect is, for many patients, itself a therapeutic event (83). This matters especially for personality pathology, where the diagnosis itself often functions as a barrier to treatment. The label personality disorder implies a verdict about character. The affective-regulatory framework carries a different message: this is how your system is currently calibrated, and calibration can change.

Psychoeducation is among the framework’s most significant clinical applications precisely because it reframes the project of therapy as building something rather than fixing something broken. Neural networks grow by addition. Extinction does not erase conditioning but builds competing inhibitory circuits alongside it (84). Recovery, in this framework, is literally construction, the progressive strengthening of cortical regulatory capacity, the incremental development of the patient’s ability to observe, modulate, contextualize, and redirect their own affective signals. This is effortful work that requires the patient’s active engagement. But it is work that can be described concretely, measured in terms of functional change, and understood by the patient as a building project with identifiable targets rather than as an open-ended exploration of something ineffably wrong with their character.

## Convergence with acceptance and commitment therapy

Of contemporary psychotherapeutic approaches, Acceptance and Commitment Therapy occupies a particularly instructive position within the affective-regulatory framework. ACT does not attempt to modify the content of primary-process affective experience. It does not ask patients to challenge the accuracy of their fear or restructure their depressive cognitions. Instead, it trains a different kind of cortical operation: the capacity to observe affective experience without being governed by it, to hold primary-process signals as events passing through consciousness rather than as imperatives demanding immediate behavioral response (85, 86).

In the language of the affective-regulatory framework, ACT builds tertiary-process regulatory capacity through a route that does not require the original conditioned association to be eliminated. This is neurobiologically sound, as again, extinction does not erase conditioning but builds new inhibitory circuits through infralimbic prefrontal and amygdala plasticity that compete with the original memory for expression (87). The six core processes of ACT can each be understood as training a specific aspect of the prefrontal regulatory system. Defusion strengthens the capacity to contextualize affective signals rather than fuse with them. Acceptance reduces compulsive regulatory maneuvers driven by SEEKING system activation, the restless affective searching that maintains pathological feedback loops. Present-moment contact and self-as-context build the observational stance that permits affective experience without automatic behavioral enactment. Committed action builds new behavioral patterns organized around cortically represented values rather than subcortical threat and reward signals.

The convergence between these two frameworks warrants attention. ACT was developed as explicitly transdiagnostic, built not for any single DSM category but for the full range of human psychological suffering, on the premise that a common set of processes underlies what the DSM carves into separate conditions. The affective-regulatory framework makes the same claim at the level of neurobiology. Both arrive at transdiagnostic conclusions from different starting points: ACT from behavioral analysis of what maintains suffering across presentations, the present framework from the neuroanatomy of affect generation and cortical regulation. That ACT arrived at this therapeutic logic without Panksepp, and the affective-regulatory framework arrived at a similar logic without Hayes, suggests that both may be approximating the same underlying reality from different angles. When a psychotherapy built on transdiagnostic behavioral theory produces effects across the same range of presentations that a transdiagnostic neurobiological framework predicts should share underlying architecture, that consilience should draw serious theoretical attention.

The contrast with cognitive behavioral therapy is instructive. CBT represented a genuine advance in its insistence on observable outcomes over untestable intrapsychic constructs (88). Aaron Beck’s empirical orientation was exactly right; what he lacked was access to the affective neuroscience that would have grounded his cognitive observations in the neural architecture producing them. The result is a treatment that works demonstrably in controlled trials, but that faces well-documented limitations precisely in the populations whose presenting problems are most rooted in deep subcortical affective dysregulation rather than surface-level cognitive distortion (89). ACT sidesteps this limitation by targeting the regulatory relationship between cortex and subcortical affect rather than the content of cognition itself.

ACT has been evaluated in 1, 447 randomized trials^1^ across a broad range of conditions (90). Preliminary neuroimaging evidence is consistent with the proposed mechanism. Following ACT intervention, participants have shown changes in functional connectivity within and between networks supporting self-reflection, emotional processing, and cognitive control, with these neural changes correlating with clinical improvement (91). That transdiagnostic reach is consistent with the present framework (92).

ACT targets the relationship between cortical regulatory systems and primary-process affective signals rather than any specific diagnostic presentation, and should therefore be applicable wherever the architecture of Affect generation and regulatory capacity is relevant. The implication is direct: if future research evaluated ACT outcomes by dimensional measures of primary-process reactivity and prefrontal regulatory capacity rather than by DSM category, we might find that the patients who benefit most are those whose affective-regulatory configuration places the greatest demand on precisely the cortical capacities ACT is designed to build.

The affective-regulatory framework generates falsifiable predictions that distinguish it from purely descriptive nosological alternatives. Pharmacological agents targeting primary-process systems should produce transdiagnostic symptom reduction across categorically distinct diagnoses sharing an affective substrate. Neuroimaging studies should demonstrate brainstem and diencephalic hyperactivation as a common correlate across affective spectrum conditions regardless of DSM category. Individuals with documented prefrontal regulatory impairment should show proportionally greater affective dysregulation across diagnostic boundaries. Dimensional affective measures should outperform categorical diagnostic assignments in predicting treatment response. These predictions are testable with existing methodologies and invite empirical engagement with the framework on its own terms.

## Limitations and competing frameworks

The framework has genuine unresolved questions that should be stated plainly. Panksepp’s most philosophically ambitious claim, that primary-process affective states are subjectively experienced, cannot be empirically demonstrated. The behavioral and neural correlates of Affect can be measured, but subjective experience cannot. The cognitive neuroscience position holds that conscious emotional experience is constructed by the cortex through integration of subcortical arousal with cognitive appraisal and conceptual categorization (93). Lisa Feldman Barrett’s constructionist framework, which is built on cognitive neuroscience, is a serious competing account with some empirical support (94). The two frameworks may capture different levels of the same system rather than being mutually exclusive, but the tension is real.

LeDoux and Brown argue that conscious emotional feelings are not generated by subcortical circuits but are higher-order cortical states, and that subcortical survival circuits operate nonconsciously, driving behavioral and physiological responses without directly producing subjective experience. Critically, they do not dispute that subcortical survival circuits drive behavior. The point of contention surrounds consciousness, which opens onto the hard problem of consciousness itself. Panksepp argued that primary-process affect is genuinely experienced at a subcortical level, while tertiary-process reflective awareness is a product of cortical processing. LeDoux collapses these into a single question and argues that because reflective awareness is cortical, feeling itself must be cortical. This debate will not be resolved soon, and for the purposes of clinical psychiatry it may not need to be. A patient is driven by the affective state regardless of where in the processing hierarchy conscious experience is ultimately located (95).

The affective-regulatory model presented here is necessarily simplified relative to the actual complexity of brain architecture. The relationship between cortical and subcortical systems involves reciprocal cortico-subcortical interactions and a well-developed cortico-thalamo-striatal loop literature that the present model does not fully incorporate. This is a genuine limitation with respect to complete neuroanatomical validity. However, for the purposes of clinical psychiatry, the core claim remains defensible: affective arousals are subcortical, the predominant direction of influence is bottom-up, and when cortical regulatory capacity is impaired, affective states persist and intensify in ways that account for a substantial domain of psychopathology. The interaction is bidirectional, and even LeDoux and Brown acknowledge that subcortical survival circuits feed upward into cortical processing while cortical circuits exert top-down modulation back onto survival circuits. The simplified model sacrifices anatomical completeness for clinical utility, and that tradeoff is both intentional and defensible.

The precision of the seven-system taxonomy is probably overstated. System boundaries are likely fuzzier and individual variation greater than the framework’s simplicity suggests. The claim that there are exactly seven primary systems rather than six or eight is almost certainly an artifact of where the research program happened to arrive.

Two existing dimensional frameworks deserve acknowledgment. The Hierarchical Taxonomy of Psychopathology correctly identifies the statistical structure of psychopathological covariance through factor analysis. The Research Domain Criteria initiative similarly aligns with the dimensional thrust of this argument. The present framework does not compete with either so much as operate at a different level, offering a mechanistic account of why the patterns HiTOP describes exist and providing anatomical and pharmacological specificity that RDoC’s valence domains only roughly approximate.

The clinical application of this framework to the large domain of psychopathology where dysregulated affect and insufficient regulatory capacity are the primary generative mechanisms draws on both theoretical reasoning and sustained clinical observation. It is offered as a set of clinical hypotheses that the model generates naturally, and that fit the pattern of what experienced clinicians encounter, not as conclusions from controlled research. Research does not yet exist in the form this framework would require.

A legitimate critique of the present argument is that it remains underdeveloped at the level of the individual primary-process systems in humans. The seven systems have not been elaborated here in the detail their formal clinical application will eventually require. How each system specifically shapes human psychopathology, and how parametric variation in each maps onto the presentations clinicians encounter, remains a program of work rather than a completed account. That is intentional. The purpose of this paper is to compile the neuroscience in order to establish the foundational architecture, not to complete the edifice. Molecular biology needed a central dogma of DNA, RNA, and protein before the mechanisms of transcription and translation could be worked out in detail. Psychiatry needs something analogous: a bottom-up organizing principle starting with subcortical Affect generation through secondary and tertiary processes, on which more granular accounts can be built. The elaboration of how each primary-process system specifically generates and shapes human psychopathology is where this work leads next.

Finally, while every patient has an affective-regulatory configuration and a personality, this framework makes no claim to explain all of psychopathology. Conditions with strong biological loading and relatively discrete genetic architecture, schizophrenia being the clearest example, require mechanistic accounts that the affective-regulatory model does not provide. The domain this framework does explain encompasses most of what practicing psychiatrists encounter in personality pathology and its common comorbidities.

## Conclusion

It is worth asking why a framework with this much converging evidence has not already transformed psychiatric practice. The researchers who built and extended Panksepp’s program are neuroscientists and psychologists. Panksepp himself trained in psychology before moving to bench neuroscience. Solms is a neuropsychologist and psychoanalyst. Montag and Davis are psychologists. The people who know this literature best do not, as a rule, practice clinical psychiatry. Harrison and Critchley identified exactly this gap in 2007, noting that Affective Neuroscience had direct implications for psychiatry that remained unaddressed (96). Nearly two decades later, the gap persists.

This paper is an attempt to close that gap, assembling the evidence for subcortical primacy of Affect for a clinical audience and applying it to the clinical domain where it is most needed. The framework describes in neurobiological terms what every practicing psychiatrist already knows: early developmental adversity sets the stage for elevated affective intensity and stunted or unbalanced cortical regulatory capacity. The insight is not new. Kraepelin’s later editions of the *Lehrbuch* included among his psychopathic personalities a category he termed *Triebmenschen*, driven persons, individuals whose behavior was governed by impulse rather than deliberation. Kraepelin described these patients as unable to resist the force of their own drives despite intact intellectual capacity. Viewed from the present vantage point, Kraepelin’s *Triebmenschen* maps with remarkable fidelity onto the affective-regulatory configuration space described here. Kraepelin intuited the right level of analysis. He simply lacked the neuroscience to specify it.

Psychoanalysis, behaviorism, and cognitive neuroscience all began from the top down. Each started with what was observable at the surface and reasoned inward. The evidence for subcortical primacy of Affect generation is now beyond compelling, and it points in the opposite direction. If psychiatry had been built bottom up around how the brain actually works rather than the canny observations of psychoanalysts, the real work could have begun sooner. It is striking that emotions occupy so little space in the DSM given that they are the foundation of personality and the primary driver of much of the suffering psychiatry exists to treat. Anger is among the most common chief complaints in clinical practice, yet the current nosology has no adequate home for it. Research into anger is correspondingly hampered by diagnostic categories that reflect neither the neuroscience nor clinical reality. It is only by fully embracing the emotional foundations of our species that the research programs needed to advance the field can be built.

In the author’s clinical practice, the affective neuroscience framework has proven genuinely useful, not as a system to be applied algorithmically, but as a conceptual foundation that makes sense of clinical complexity and that gives patients a more accurate and more empowering account of their own inner lives. Patients who understand that their suffering has an identifiable architecture, and that the project of recovery is one of building regulatory capacity rather than overcoming a character flaw, engage differently in treatment. That is the test that matters most, not whether the framework is theoretically elegant, but whether it guides clinicians toward more accurate understanding and patients toward more effective recovery.

## Data Availability

The original contributions presented in the study are included in the article/supplementary material, further inquiries can be directed to the corresponding author.

## Footnotes

1 Association for Contextual Behavioral Science. ACT randomized controlled trials (1986 to present). (2026). Available at: https://contextualscience.org/ACT_Randomized_Controlled_Trials.
